# Dual-route processing of horizontally and vertically jumbled Chinese words: evidence from ND250 and LAN

**DOI:** 10.3389/fpsyg.2026.1873297

**Published:** 2026-08-28

**Authors:** Axu Hu, Shu Liu, Feng Gu

**Affiliations:** 1 Key Laboratory of Language and Cultural Computing of Ministry of Education, Northwest Minzu University, Lanzhou, China; 2 Neurocognitive Laboratory for Linguistics and Semiotics, College of Chinese Language and Literature, Sichuan University, Chengdu, China; 3 Digital Convergence Laboratory of Chinese Cultural Inheritance and Global Communication, Sichuan University, Chengdu, China

**Keywords:** Chinese words, compound words, LAN, ND250, visual word recognition

## Abstract

Previous research suggests that visual recognition of two-character Chinese compound words relies on a dual-route mechanism involving whole-word orthographic processing and morphological decomposition, both of which remain engaged for horizontally presented jumbled words. The present study investigated whether this flexible dual-route mechanism generalizes to vertically presented jumbled words and whether the two processing routes differ in their sensitivity to presentation orientation (horizontal vs. vertical) and character order (canonical vs. jumbled). Event-related potentials (ERPs) were measured during an implicit reading task, in which two-character Chinese words and pseudowords were presented under four conditions: horizontal canonical, horizontal jumbled, vertical canonical, and vertical jumbled. Both ND250 and left anterior negativity (LAN) effects were reliably elicited across all four conditions, indicating that both processing routes remain engaged across presentation orientations and character orders. ND250 mean amplitude showed an interaction between presentation orientation and character order, and ND250 peak latency was affected by presentation orientation, indicating that whole-word orthographic processing is sensitive to visuospatial organization. In contrast, neither LAN amplitude nor peak latency was affected by either factor, suggesting that decomposition-related processing is relatively stable across spatial configurations.

## 1 Introduction

Reading is a fundamental cognitive skill in modern society. Skilled readers can rapidly access phonological and semantic information from a vast number of written words within a few 100 milliseconds, reflecting the remarkable computational capacity of the human brain. Crucially, visual word recognition is flexible, allowing readers to recognize visually distorted words even when their visual structure is disrupted. A classic example is the transposed-letter (or jumbled word) effect (e.g., Rayner et al., 2006; Johnson et al., 2007), whereby words remain readable even when their internal letter order is disrupted—for example, “it deosn’t mttaer in waht oredr the ltteers in a wrod are.” Extensive research has shown that this effect is robust but sensitive to the nature of letter-position disruption. Jumbled words are generally more difficult to process than their canonical forms, particularly when external or nonadjacent letters are transposed (e.g., Rayner et al., 2006; Perea et al., 2008; White et al., 2008). Converging evidence from behavioral and electrophysiological research suggests that the transposed-letter effect arises because jumbled words activate the lexical representations of their corresponding base words (e.g., Grainger et al., 2006; Carreiras et al., 2007; Johnson et al., 2007; Carreiras et al., 2009). A comparable phenomenon has also been documented in Chinese, where words are composed of characters rather than letters. This phenomenon is commonly referred to as the transposed-character effect and reflects flexible encoding of character position in Chinese word recognition (e.g., Gu et al., 2015; Chang et al., 2020; Lin and Lin, 2020; Zhang et al., 2022; Gu et al., 2023; Sun and Pae, 2024). Similar to the transposed-letter effect, the transposed-character effect in Chinese is also constrained by visuospatial factors, such as transposition distance and intercharacter spacing (e.g., Zhang et al., 2022; Gu et al., 2023). Importantly, however, most Chinese words are compounds whose constituent characters correspond to meaningful morphemes (Zhou et al., 1999). Thus, transposing characters in Chinese typically also entails transposing constituent morphemes within a compound word. This raises an important question regarding how Chinese compound words are processed when the spatial arrangement of their constituent morphemes is disrupted.

The recognition of compound words has been proposed to rely on two processing routes (Kuperman et al., 2009; Beyersmann et al., 2012; Li et al., 2026): a whole-word route, in which the compound is processed as a holistic lexical unit, and a decomposition route, in which the compound is processed via its constituent morphemes. In event-related potentials (ERP) research, two components have been closely linked to these processing routes. One is the ND250, which typically emerges over occipito-temporal scalp regions roughly 250 ms after stimulus onset and is sensitive to lexicality and word frequency, showing stronger negative responses to pseudowords than to real words, and to low-frequency words than to high-frequency words (e.g., Yu et al., 2022; Zhang et al., 2023; Ran et al., 2026). This component is generally interpreted as reflecting automatic processing of visual word forms, although its precise neural mechanisms remain unclear (Liu et al., 2025; Huang et al., 2026). Notably, the ND250 has also been reported for Chinese compound words, suggesting that such stimuli are processed as whole units (Huang et al., 2023; Hu et al., 2026). The other component is the left anterior negativity (LAN), which is typically observed over left anterior scalp regions around 400–450 ms after stimulus onset. The LAN has been widely associated with the processing of morphologically complex words and is often linked to decomposition-related mechanisms, such as the analysis of constituent morphemes and their integration into a coherent representation (for a review, see Leminen et al., 2019). Together, these components provide complementary neural markers for investigating whole-word orthographic processing and decomposition-related processing during Chinese compound-word recognition. Using these ERP components, Hu et al. (2026) investigated whether both processing routes are engaged during the recognition of Chinese jumbled words. Participants performed an implicit color-detection task while viewing two-character words, jumbled words, and pseudowords under two horizontal presentation conditions. In the canonical condition, the two constituent characters were presented in their conventional left-to-right order, whereas in the jumbled condition, their order was reversed (AB → BA). Both canonical and jumbled words elicited significant ND250 and LAN effects relative to pseudowords. Thus, Hu et al. (2026) provided electrophysiological evidence that both whole-word and decomposition-related processing contribute to the recognition of horizontally presented Chinese jumbled words.

**Figure 1:**
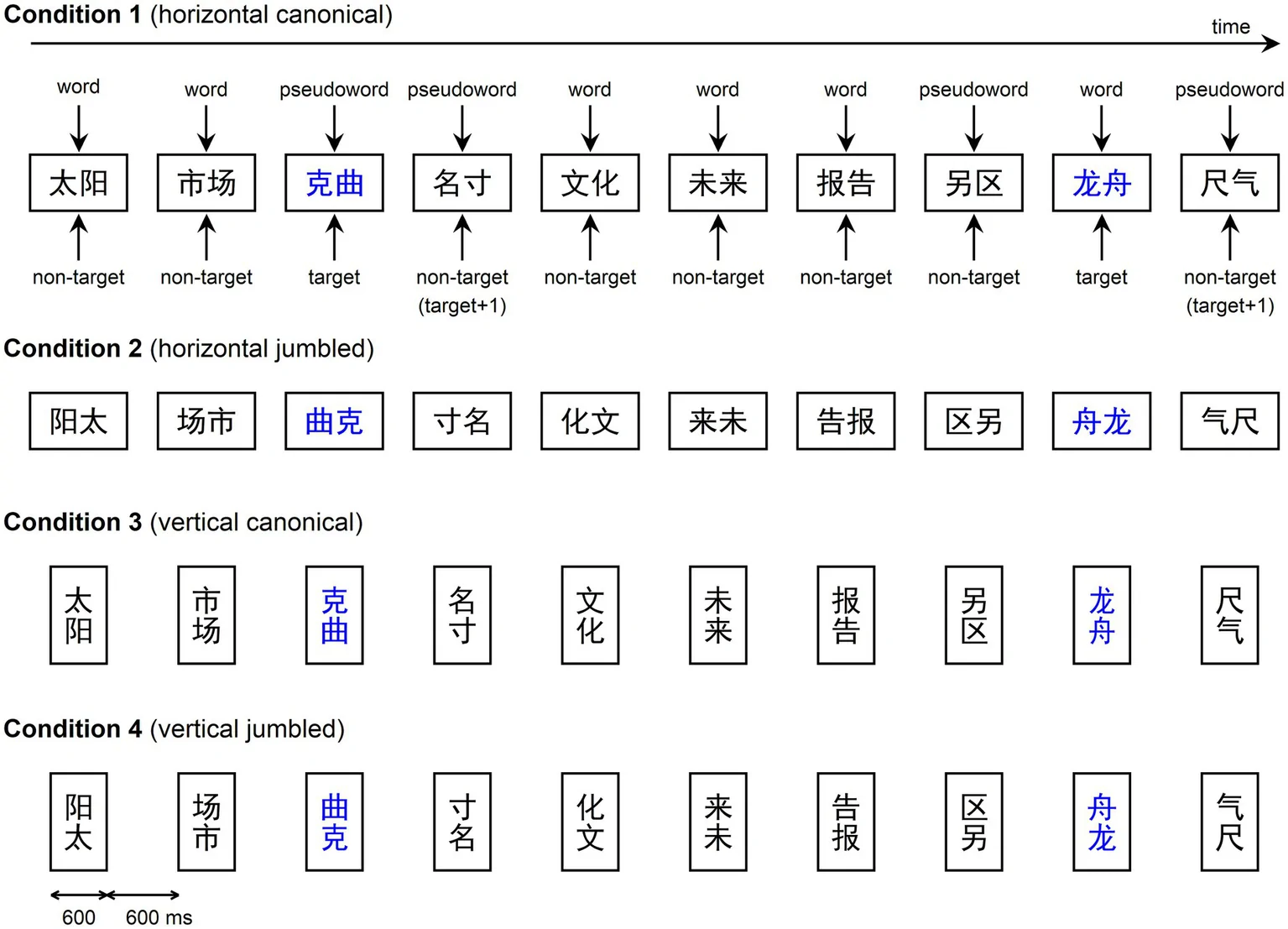
Experimental conditions and general trial procedure. The experiment included four conditions defined by Presentation Orientation and Character Order: Condition 1 (horizontal canonical), Condition 2 (horizontal jumbled), Condition 3 (vertical canonical), and Condition 4 (vertical jumbled). Across all conditions, stimuli were presented for 600 ms followed by a 600-ms blank interval. Participants responded only to blue targets while ignoring lexical content. Non-target trials immediately following target trials were designated as “target+1” trials; both target and “target+1” trials were excluded from ERP averaging to minimize response-related contamination.

Modern Chinese is typically written from left to right, similar to English. However, vertical writing formats arranged from top to bottom are also widely used in contemporary contexts, such as signage, calligraphic works, public plaques, and book titles. Examining vertically presented Chinese compound words is therefore important for assessing whether the mechanisms observed in horizontal reading also operate in this commonly encountered alternative writing format. Previous behavioral research has shown that the transposed-character effect can also be observed under vertical presentation, indicating that disruption of vertical character order does not necessarily prevent successful word recognition (Yang et al., 2019). However, it remains unclear whether vertically presented jumbled words engage the same whole-word and decomposition-related processing mechanisms as horizontally presented jumbled words, and whether these two processing routes differ in their sensitivity to presentation orientation and character order. To address these questions, the present study orthogonally manipulated Presentation Orientation (horizontal vs. vertical) and Character Order (canonical vs. jumbled), extending the horizontal canonical and horizontal jumbled conditions used in Hu et al. (2026) to include vertical canonical and vertical jumbled conditions. Following Hu et al. (2026), an implicit target detection task was employed in which participants responded only to stimulus color while ignoring lexical content (Figure 1). This task was chosen to examine the automatic processing of real words, jumbled words, and pseudowords while minimizing explicit lexical-semantic decision demands. Stimulus color served only as the task-relevant dimension, whereas contrasts between words/jumbled words and pseudowords were used to derive the ND250 and LAN effects. This design allowed whole-word orthographic processing and decomposition-related processing to be examined under different spatial configurations without requiring explicit lexical judgments.

The present study aimed to address three research questions (RQs):

*RQ1*: Do vertically presented jumbled Chinese words engage both whole-word orthographic processing and decomposition-related processing, as reflected by the presence of ND250 and LAN effects?

*RQ2*: Is whole-word orthographic processing sensitive to Presentation Orientation and Character Order, as reflected by ND250 mean amplitude and peak latency?

*RQ3*: Is decomposition-related processing sensitive to Presentation Orientation and Character Order, as reflected by LAN mean amplitude and peak latency?

Based on Hu et al. (2026), vertically jumbled words were expected to elicit both ND250 and LAN effects, similar to horizontally presented jumbled words (RQ1). For the analyses addressing RQ2 and RQ3, component amplitude was taken to reflect the magnitude of the neural processing indexed by each ERP component, whereas peak latency was taken to reflect its temporal dynamics. Thus, ND250 amplitude and latency were used to assess the magnitude and timing of whole-word orthographic processing, respectively, whereas LAN amplitude and latency were used to assess the corresponding aspects of decomposition-related processing. Because little direct evidence is available regarding how presentation orientation and character order affect whole-word orthographic processing and decomposition-related processing, no directional predictions were made for RQ2 and RQ3; these analyses were therefore exploratory. Together, these research questions were intended to clarify the mechanisms underlying the flexibility of Chinese visual word recognition and to refine the dual-route framework of Chinese compound-word recognition by determining whether the whole-word and decomposition-related routes differ in their sensitivity to changes in presentation orientation and character order.

## 2 Materials and methods

### 2.1 Participants

An *a priori* power analysis was performed using G*Power (version 3.1) to determine the required sample size for the statistical analyses corresponding to the three research questions. For RQ1, the presence of ND250 and LAN effects within each condition was assessed using one-sample *t*-tests against zero. Because the effects were tested across four conditions, a Bonferroni-adjusted alpha level of 0.0125 (0.05/4) was used. Assuming a medium effect size (Cohen’s *d* = 0.5), a statistical power of 0.80, and a two-tailed test, the minimum required sample size was estimated to be 48 participants. For RQ2 and RQ3, the effects of Presentation Orientation and Character Order were assessed using 2 × 2 repeated-measures ANOVAs, followed by paired-samples *t*-tests when significant interactions were observed. An *a priori* power analysis for the repeated-measures ANOVA, assuming a medium effect size (Cohen’s *f* = 0.25), α = 0.05, and power = 0.80, indicated a minimum required sample size of 24 participants. Because significant interactions were followed by two pairwise comparisons with Bonferroni correction, an additional power analysis for a two-tailed paired-samples *t*-test was conducted using a medium effect size (Cohen’s *d* = 0.5), a Bonferroni-adjusted α of 0.025 (0.05/2), and power = 0.80, indicating a minimum required sample size of 41 participants. To allow for potential data loss arising from EEG artifact rejection, a total of 52 participants were initially recruited. Following data preprocessing, four participants were excluded due to excessive EEG artifacts, yielding a final sample of 48 native Chinese readers (27 females, 21 males; aged 19–27 years, *M* = 21.5, *SD* = 2.0). Thus, the final sample met or exceeded the sample-size requirements for all planned analyses.

All participants were students at Sichuan University with normal or corrected-to-normal vision. None reported any history of reading impairments or neurological conditions. Handedness was evaluated using the Edinburgh Handedness Inventory (Oldfield, 1971), and all participants were confirmed to be right-handed. All participants gave written informed consent, and the study was approved by the Ethics Committee of the College of Chinese Language and Literature at Sichuan University.

### 2.2 Stimuli

The stimuli were identical to those used in Hu et al. (2026), although all behavioral and EEG data were newly collected from an independent sample for the present study, which extended the two horizontal conditions of Hu et al. (2026) to a four-condition design by additionally manipulating presentation orientation. In brief, the non-target stimuli, excluding “target+1” trials (see Figure 1), included 120 words and 120 pseudowords. The words were selected to be high in frequency and visually simple. The real words had a mean word frequency of 120.3 ± 443.4 occurrences per million and a mean stroke count of 10.9 ± 1.7 per word (*M* ± *SD*). Additional constraints were applied to minimize visual and lexical confounds, including the exclusion of reversible words and repeated-character words, while emotionally salient items were kept to a minimum to reduce the potential influence of emotional content on the ERP results (Zhang et al., 2024).

The 120 pseudowords were created by rearranging the constituent characters of the 120 real two-character words to generate novel character combinations that were not present in the lexicon. Because the same character inventory was used for both real words and pseudowords, the two stimulus types shared identical constituent characters, allowing character-level visual properties to be controlled while lexical status was manipulated. For the jumbled conditions, the two constituent characters of each word or pseudoword were presented in reversed order (AB → BA), resulting in non-canonical character sequences. All pseudowords and their transposed forms were checked against the Chinese Lexical Database (Sun et al., 2018) to ensure that they did not correspond to existing lexical items.

An additional set comprising 30 words and 30 pseudowords was generated according to the same criteria, which were used in target and “target+1” trials. These trials were excluded from the EEG analyses to minimize contamination from response-related and residual post-response activity. Full details of stimulus construction and linguistic characteristics are provided in Hu et al. (2026).

### 2.3 Procedure

Participants sat approximately 135 cm from the display. Prior to the main experiment, they completed a short practice block with stimuli not used in the main experiment. During the experiment, participants performed a target-detection task in which they were instructed to make a speeded and accurate keypress whenever a stimulus appeared in blue and to withhold responses to stimuli presented in black. Participants were not required to judge the lexical status or meaning of the stimuli. Thus, real words, jumbled words, and pseudowords were task-irrelevant with respect to the overt response, and only stimulus color determined whether a response was required. Responses were made using the index finger, with the response hand (left vs. right) counterbalanced across participants.

The general trial procedure was identical across all experimental conditions. Each stimulus was presented centrally in Heiti font against a white background for 600 ms, followed by a blank white screen for 600 ms, yielding a stimulus onset asynchrony of 1,200 ms. Responses were recorded during a 2,000-ms window beginning at stimulus onset, and a response did not terminate the trial or alter the fixed presentation sequence. Each condition comprised 600 trials, including 60 blue target trials requiring a keypress and 540 black non-target trials. Among the non-target trials, 60 were “target+1” trials, defined as the trials immediately following target trials. Target and “target+1” trials were excluded from the ERP analyses to minimize contamination from response-related and residual post-response activity. No more than three consecutive trials contained stimuli from the same lexical category (word or pseudoword).

The experiment comprised four conditions that differed only in Presentation Orientation and Character Order (Figure 1). Presentation Orientation was either horizontal or vertical, and Character Order was either canonical or jumbled, resulting in Condition 1 (horizontal canonical), Condition 2 (horizontal jumbled), Condition 3 (vertical canonical), and Condition 4 (vertical jumbled). Specifically, the two constituent characters were arranged from left to right in Condition 1, right to left in Condition 2, top to bottom in Condition 3, and bottom to top in Condition 4. The order of the four conditions was counterbalanced across participants using a Latin square design.

The same set of 300 stimuli—150 words and 150 pseudowords—was used in each condition and presented twice in different pseudorandom orders, resulting in 600 trials per condition. Short breaks were provided between conditions. Stimulus presentation and response recording were controlled using E-Prime 3.0.

### 2.4 EEG recording

Continuous EEG data were recorded using a 64-channel elastic cap with Ag/AgCl electrodes arranged according to the international 10–10 system. Vertical electrooculograms (VEOGs) were monitored using a pair of bipolar electrodes positioned above and below the left eye. The nose tip served as the reference, and AFz was used as the ground. Electrode impedances were kept below 5 kΩ throughout the recording. EEG signals were amplified using a SynAmps 2 system (Compumedics Neuroscan), with an online bandpass filter of 0.05–200 Hz and a sampling rate of 500 Hz.

### 2.5 Data analysis

#### 2.5.1 EEG preprocessing

For each participant, the continuous EEG data were digitally band-pass filtered between 0.1 and 25 Hz. Ocular artifacts associated with eye blinks were corrected using the regression-based procedure proposed by Semlitsch et al. (1986). The filtered data were then segmented into epochs ranging from −100 to 500 ms relative to stimulus onset. Target and “target+1” trials were excluded from further analyses to minimize contamination from response-related and residual post-response activity. Baseline correction was applied using the 100 ms pre-stimulus interval, after which epochs exceeding ±50 μV at any electrode (excluding VEOG) were rejected. After artifact rejection, the mean numbers of retained epochs per participant were 185.7 ± 33.8 for words and 187.6 ± 30.5 for pseudowords in Condition 1, 196.0 ± 29.5 for words and 195.2 ± 30.7 for pseudowords in Condition 2, 193.2 ± 31.2 for words and 195.1 ± 31.0 for pseudowords in Condition 3, and 190.3 ± 28.9 for words and 191.8 ± 28.0 for pseudowords in Condition 4, respectively. The remaining epochs were averaged separately for each condition and stimulus type (words or jumbled words vs. pseudowords) to obtain ERP waveforms, which were then re-referenced to the common average of the 64 scalp electrodes.

#### 2.5.2 Statistical analysis of ND250 and LAN effects

For each participant and condition, ND250 difference waves were computed as pseudowords minus words or jumbled words. Analyses were conducted over a left occipito-temporal cluster including P7, P5, PO7, PO5, and O1. Mean amplitudes were extracted from the 240–300 ms interval and averaged across these electrodes before statistical testing. Peak latency was defined at PO7 as the latency of the most negative deflection within the ND250 time range. This electrode cluster and temporal window were selected based on previous evidence that ND250 responses to Chinese compound words are maximal over left occipito-temporal regions within this interval (Huang et al., 2023; Hu et al., 2026).

For each participant and condition, LAN difference waves were computed as words or jumbled words minus pseudowords. Analyses were conducted over a left anterior electrode cluster including FP1, AF3, F7, F5, and F3. Mean amplitudes were extracted from the 400–460 ms interval and averaged across these electrodes before statistical testing. Peak latency was defined at FP1 as the latency of the most negative deflection within the LAN time range. This electrode cluster and temporal window were selected based on previous evidence that LAN responses associated with Chinese compound word processing are maximal over left anterior regions within this interval (Wei et al., 2023; Hu et al., 2026).

## 3 Results

### 3.1 Behavioral results

Target detection accuracy was computed for each participant. Performance was near ceiling, with mean accuracies of 99.62, 99.48, 99.48, and 99.31% across the four conditions, respectively, indicating that participants complied closely with the task requirements. Mean response times (± *SD*) were 418 ± 51 ms, 421 ± 45 ms, 423 ± 46 ms, and 418 ± 44 ms for the four conditions, respectively. To examine whether response times differed across conditions, a repeated-measures ANOVA with Presentation Orientation (horizontal vs. vertical) and Character Order (canonical vs. jumbled) as within-subject factors revealed no significant main effects of Presentation Orientation, *F*(1, 47) = 0.075, *p* = 0.785, or Character Order, *F*(1, 47) = 0.089, *p* = 0.766, nor a significant interaction between the two factors, *F*(1, 47) = 1.58, *p* = 0.214.

### 3.2 ERP results

**Figure 2:**
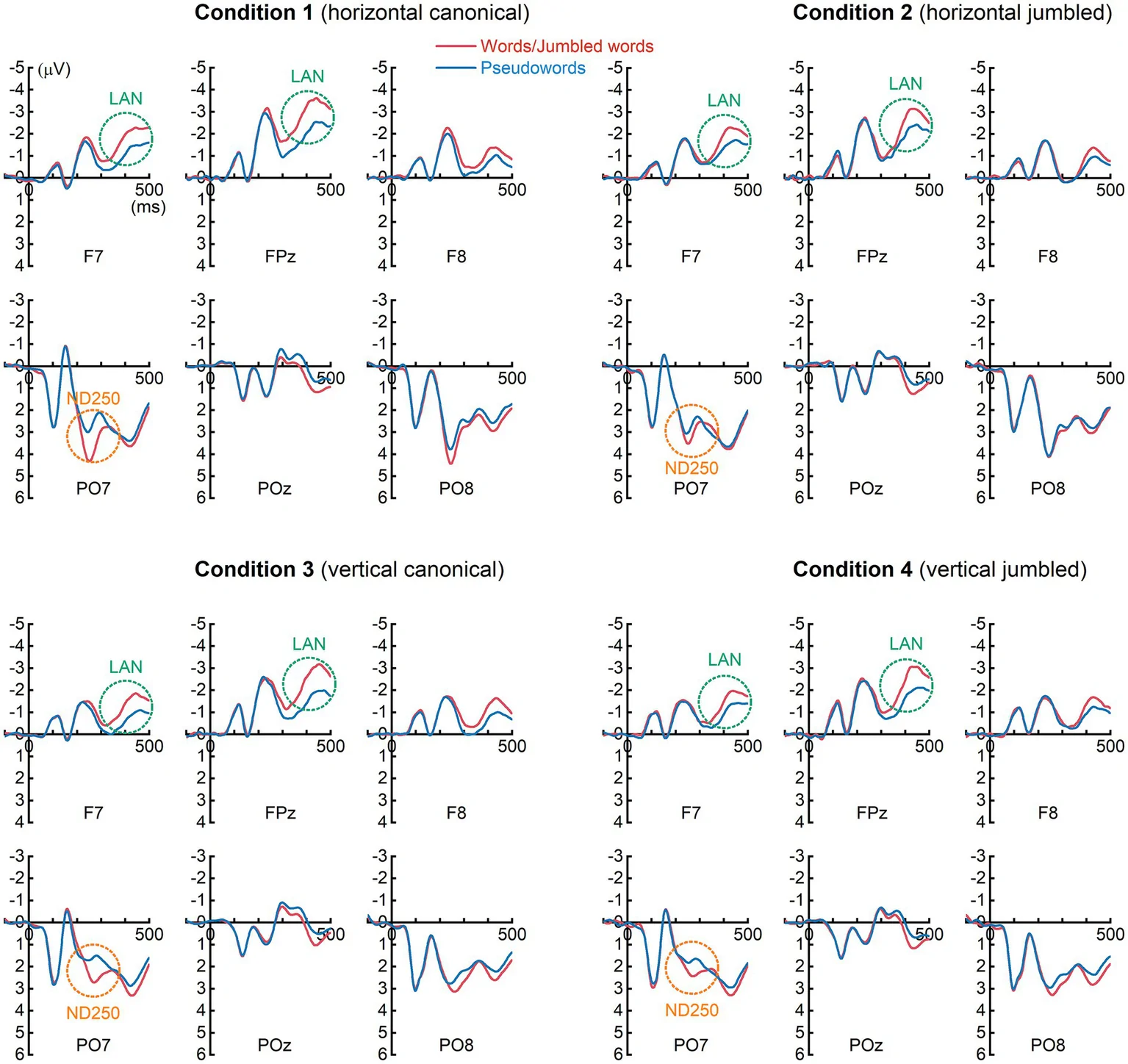
Grand-averaged ERP waveforms elicited by words/jumbled words and pseudowords across the four experimental conditions. The ND250 pattern is shown at PO7, where pseudowords produced stronger negative-going activity than words/jumbled words, and is highlighted by orange dotted circles. The LAN pattern is shown at F7 and FPz, where words/jumbled words produced stronger negative-going activity than pseudowords, and is highlighted by green dotted circles.

As shown in Figure 2, the ERP waveforms differed systematically between words/jumbled words and pseudowords across the four conditions. At the posterior electrode PO7, pseudowords elicited stronger negativities than words/jumbled words, corresponding to the ND250 effect highlighted by the orange dotted circles. Over left anterior sites, particularly F7 and FPz, the opposite pattern was observed: words/jumbled words elicited stronger negativities than pseudowords, corresponding to the LAN effect highlighted by the green dotted circles.

**Figure 3:**
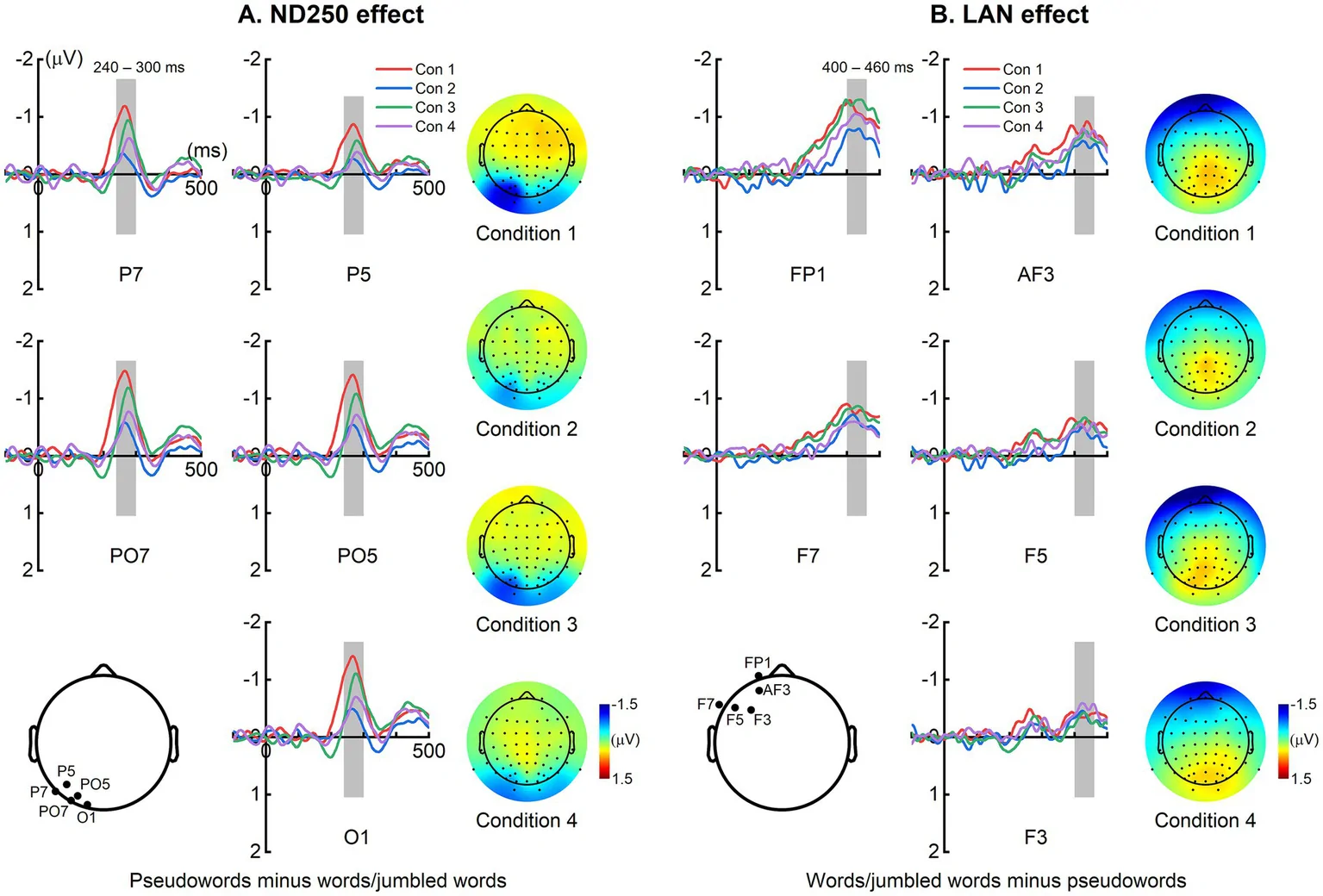
Grand-averaged difference waveforms and scalp topographies for the ND250 and LAN effects across the four experimental conditions. **(A)** ND250 difference waves were computed as pseudowords minus words/jumbled words and are displayed over posterior electrodes (P7, P5, PO7, PO5, O1). **(B)** LAN difference waves were computed as words/jumbled words minus pseudowords and are displayed over anterior electrodes (FP1, AF3, F7, F5, F3). Gray shaded regions indicate the analysis windows for mean amplitude quantification: 240–300 ms for the ND250 and 400–460 ms for the LAN. Topographies show the scalp distribution of each effect by condition.

**Figure 4:**
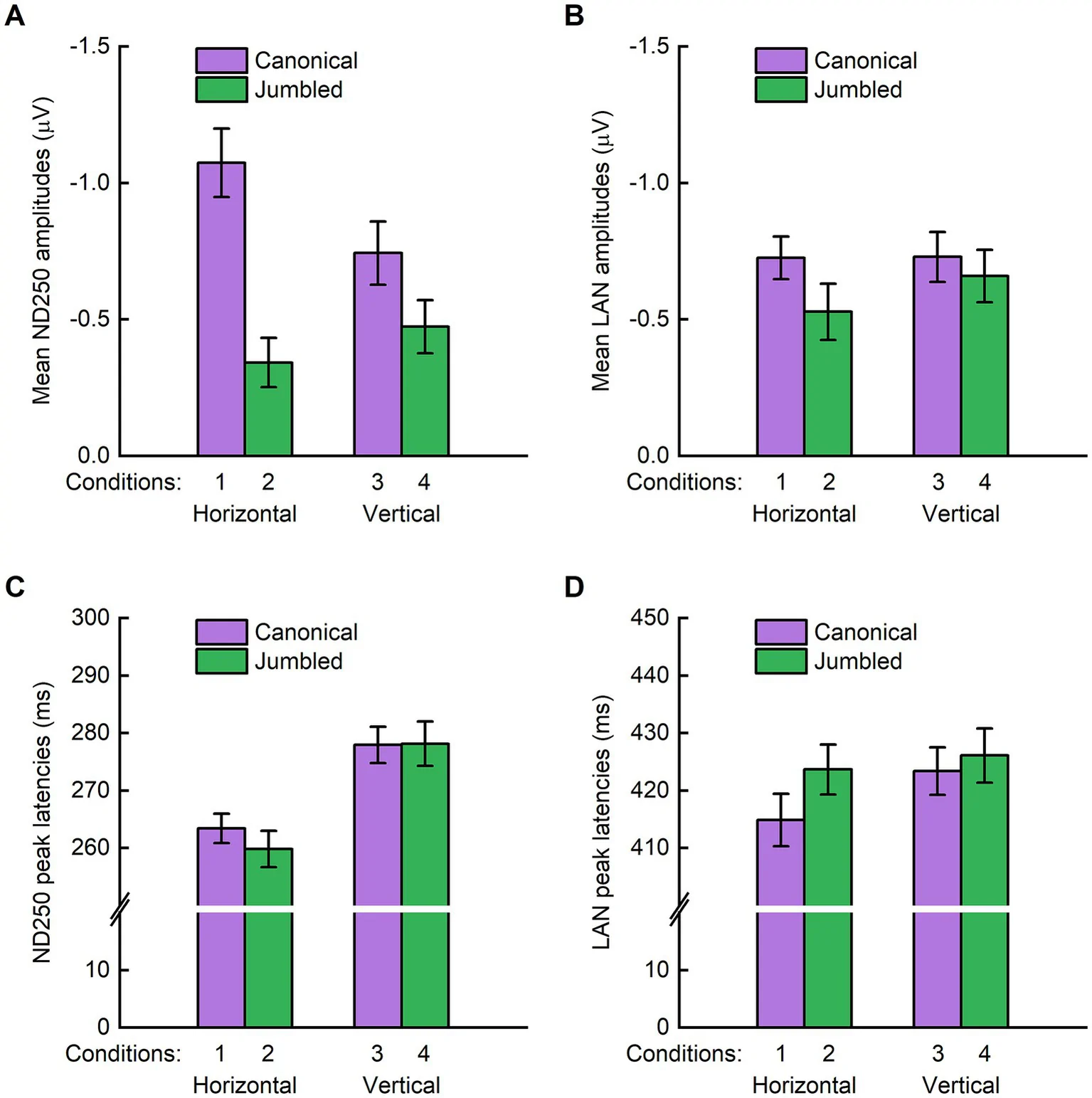
Mean amplitudes and peak latencies of the ND250 and LAN effects across conditions. **(A)** Mean ND250 amplitudes (240–300 ms) at posterior electrodes (P7, P5, PO7, PO5, and O1). A significant interaction between Presentation Orientation and Character Order was observed, with a larger ND250 difference between horizontal and vertical conditions in the canonical condition than in the jumbled condition. **(B)** Mean LAN amplitudes (400–460 ms) at frontal electrodes (FP1, AF3, F7, F5, and F3). No significant effects of Presentation Orientation or Character Order, nor their interaction, were observed. **(C)** Peak latencies of the ND250. A significant main effect of Presentation Orientation was observed, with later peaks for vertical than horizontal conditions, whereas no effect of Character Order or interaction was found. **(D)** Peak latencies of the LAN. No significant effects of Presentation Orientation or Character Order, nor their interaction, were observed. Error bars represent ±1 SEM.

To address RQ1, whether vertically presented jumbled Chinese words engage both whole-word orthographic processing and decomposition-related processing was examined by testing the presence of the ND250 and LAN effects across the four experimental conditions. The ND250 effect, computed by subtracting the ERPs to words/jumbled words from those to pseudowords, at the electrodes selected for mean amplitude analysis (P7, P5, PO7, PO5, and O1) is shown in Figure 3A. The corresponding scalp distributions of the ND250 within the 240–300 ms time window are presented for each condition, and the mean amplitude results are shown in Figure 4A. The ND250 effect was significant in all four conditions, as confirmed by one-sample *t*-tests against zero with Bonferroni correction for four comparisons [Condition 1: *t*(47) = 8.56, *p* < 0.001, two-tailed; Condition 2: *t*(47) = 3.80, *p* = 0.002, two-tailed; Condition 3: *t*(47) = 6.43, *p* < 0.001, two-tailed; Condition 4: *t*(47) = 4.87, *p* < 0.001, two-tailed]. The LAN effect, computed by subtracting the ERPs to pseudowords from those to words/jumbled words, at the electrodes selected for mean amplitude analysis (FP1, AF3, F7, F5, and F3) is shown in Figure 3B. The corresponding scalp distributions of the LAN within the 400–460 ms time window are presented for each condition, and the mean amplitude results are shown in Figure 4B. The LAN effect was significant in all four conditions, as confirmed by one-sample *t*-tests against zero with Bonferroni correction for four comparisons [Condition 1: *t*(47) = 9.25, *p* < 0.001, two-tailed; Condition 2: *t*(47) = 5.11, *p* < 0.001, two-tailed; Condition 3: *t*(47) = 7.98, *p* < 0.001, two-tailed; Condition 4: *t*(47) = 6.86, *p* < 0.001, two-tailed].

To address RQ2, whether whole-word orthographic processing is sensitive to Presentation Orientation and Character Order was examined using ND250 mean amplitude and peak latency, which were analyzed with repeated-measures ANOVAs. For mean amplitude, a significant interaction between the two factors was observed, *F*(1, 47) = 8.02, *p* = 0.007. Follow-up paired-samples *t*-tests were conducted to examine the source of this interaction. After Bonferroni correction for the two comparisons, the interaction was driven by a significant difference in ND250 amplitude between Condition 1 (horizontal canonical) and Condition 3 (vertical canonical), *t*(47) = 2.61, *p* = 0.024 (Bonferroni-adjusted), whereas no significant difference was found between Condition 2 (horizontal jumbled) and Condition 4 (vertical jumbled), *t*(47) = −1.20, *p* = 0.470 (Bonferroni-adjusted). For peak latency (Figure 4C), a repeated-measures ANOVA revealed a significant main effect of Presentation Orientation, *F*(1, 47) = 37.23, *p* < 0.001. Specifically, the ND250 peaked later for vertically presented stimuli (*M* = 278.0 ms, *SD* = 17.4) than for horizontally presented stimuli (*M* = 261.6 ms, *SD* = 15.7), with values collapsed across Character Order conditions. No significant main effect of Character Order was observed, *F*(1, 47) = 0.28, *p* = 0.597, nor a significant interaction between the two factors, *F*(1, 47) = 0.43, *p* = 0.517.

To address RQ3, whether decomposition-related processing is sensitive to Presentation Orientation and Character Order was examined using LAN mean amplitude and peak latency, which were analyzed with repeated-measures ANOVAs. For mean amplitude (Figure 4B), no significant main effects or interaction were observed (*p*s > 0.209). Similarly, for peak latency (Figure 4D), no significant main effects or interaction were found (*p*s > 0.207).

## 4 Discussion

The present study investigated the processing of horizontally and vertically presented Chinese jumbled words within a dual-route framework of compound-word recognition. Robust ND250 and LAN effects were observed across all four conditions, including the vertical jumbled condition, indicating that both whole-word orthographic processing and decomposition-related processing remain engaged when the spatial arrangement of constituent morphemes is disrupted. Furthermore, the ND250 amplitude and latency results revealed that whole-word orthographic processing is sensitive to visuospatial organization, whereas the absence of significant effects on LAN amplitude and latency suggests that decomposition-related processing remains comparatively stable across different spatial configurations. Together, these findings demonstrate that both processing routes contribute to Chinese compound-word recognition under non-canonical spatial arrangements, but differ in their sensitivity to presentation orientation and character order.

### 4.1 Flexible engagement of dual routes in Chinese compound-word recognition

Addressing RQ1, the present study examined whether both whole-word orthographic processing and decomposition-related processing remained engaged when Chinese compound words were presented under altered spatial configurations, as indexed by the ND250 and LAN, respectively.

The ND250 is considered an electrophysiological marker of lexical-level orthographic processing of visual word forms and is associated with activity in the ventral occipitotemporal cortex (e.g., Yu et al., 2022; Zhang et al., 2023; Liu et al., 2025; Huang et al., 2026; Ran et al., 2026). Previous studies have shown that Chinese compound words elicit ND250 effects, suggesting that they can be processed as whole lexical units (Huang et al., 2023). Hu et al. (2026) further demonstrated that horizontally jumbled Chinese words also elicit ND250 effects, indicating partial activation of whole-word orthographic representations despite disrupted character order. Extending these findings, the present study observed robust ND250 effects for vertically presented jumbled words (Figures 2–4), demonstrating that whole-word orthographic representations remain accessible even when both presentation orientation and character order are altered. The present findings suggest that the whole-word processing route in Chinese compound-word recognition may rely on a flexible character position coding mechanism analogous to letter position coding mechanisms in alphabetic languages. Numerous computational models, such as the SERIOL model (Whitney, 2001), the open-bigram model (Grainger and Van Heuven, 2004), and the spatial coding model (Davis, 2010), have been proposed to explain the flexibility of letter position coding underlying jumbled word processing in alphabetic languages. Although comparable models for Chinese compound-word processing remain limited, the present findings provide important constraints for future models by demonstrating that whole-word orthographic representations should incorporate flexible spatial representations of constituent characters, allowing access to these representations despite non-canonical configurations.

The LAN has been associated with decomposition-related processing, including the analysis of constituent morphemes and their integration into a coherent representation (for a review, see Leminen et al., 2019). In Chinese, where individual characters typically correspond to meaningful morphemes, LAN effects have been interpreted as reflecting the involvement of morpheme-based processing during compound-word recognition. Consistent with this interpretation, Hu et al. (2026) showed that Chinese compound words elicit LAN effects and that such effects remain present for horizontally jumbled words, suggesting that decomposition-related processing can proceed despite disrupted character order. Extending these findings, the present study demonstrated robust LAN effects across all four conditions (Figures 2–4), including vertically presented jumbled words.

Together, the ND250 and LAN findings indicate that both whole-word orthographic processing and decomposition-related processing remain engaged when the spatial arrangement of constituent characters is disrupted. Thus, Chinese compound-word recognition does not depend exclusively on canonical configurations. Instead, both processing routes can contribute to word recognition under non-canonical spatial arrangements, although their sensitivity to visuospatial organization differs (see Sections 4.2 and 4.3).

### 4.2 ND250 reflects the visuospatial sensitivity of whole-word orthographic processing in Chinese compound-word recognition

Having established that whole-word orthographic processing remains engaged under altered spatial configurations, we next addressed RQ2 by examining whether this processing is modulated by presentation orientation and character order.

The ND250 mean amplitude results revealed a significant interaction between Presentation Orientation and Character Order (Figure 4A). This interaction may reflect experience-dependent tuning of orthographic representations. In modern Chinese, horizontal presentation represents the dominant writing format, making horizontally presented canonical words (Condition 1) the most familiar configuration. Although less frequent than horizontal presentation, vertical canonical words (Condition 3) are still encountered in certain contexts and may therefore retain a certain degree of familiarity. In contrast, jumbled words presented in either horizontal or vertical formats (Conditions 2 and 4) represent highly unfamiliar orthographic configurations because such character arrangements rarely occur in everyday reading. The modulation of ND250 amplitude appears to reflect these differences in the familiarity of orthographic configurations, with more familiar configurations eliciting stronger whole-word orthographic engagement. Importantly, although jumbled words are rarely encountered outside experimental contexts, they still elicited robust ND250 effects, indicating that whole-word orthographic representations can be partially accessed even when the canonical spatial arrangement of constituent characters is disrupted.

The ND250 peak latency showed a different pattern, with a significant main effect of Presentation Orientation but no effect of Character Order or interaction (Figure 4C). Specifically, ND250 peaks were delayed for vertically presented stimuli relative to horizontally presented stimuli, regardless of whether the character order was canonical or jumbled. This pattern suggests that presentation orientation primarily influences the temporal dynamics of whole-word orthographic access, whereas character order does not substantially alter the timing of this process. Because horizontal presentation represents the dominant format of modern Chinese reading, horizontally presented stimuli, irrespective of character order (i.e., canonical or jumbled), may benefit from more efficient perceptual-to-orthographic mapping, whereas vertically presented stimuli, also irrespective of character order, may require additional processing time before whole-word orthographic representations are accessed.

Together, the amplitude and latency findings suggest that different aspects of the ND250 capture partially distinct influences of visuospatial organization on whole-word processing. ND250 amplitude appears to reflect variations in the degree of whole-word orthographic engagement under different combinations of presentation orientation and character order, whereas ND250 latency reflects differences in the temporal efficiency of accessing whole-word orthographic representations across presentation formats.

### 4.3 LAN reflects relatively stable decomposition-related processing in Chinese compound-word recognition

Having established that decomposition-related processing remains engaged under altered spatial configurations (see Section 4.1), we next addressed RQ3 by examining whether this processing is modulated by presentation orientation and character order.

Neither LAN mean amplitude nor peak latency was significantly affected by Presentation Orientation or Character Order (Figures 4B,D). This stability suggests that LAN-related processing is relatively insensitive to changes in the spatial configuration of constituent characters, remaining robust across different presentation orientations and character orders. This pattern suggests that constituent morphemes remain sufficiently identifiable despite changes in their relative positions, allowing decomposition- and integration-related processing to proceed in a relatively stable manner. Unlike whole-word orthographic processing, which may depend more strongly on learned visual configurations of complete word forms, decomposition-related processing may rely on representations of constituent morphemes that are less dependent on their exact spatial arrangement.

The relative stability of the LAN may reflect the recruitment of more general mechanisms involved in integrating meaningful visual elements. Reading is a culturally acquired skill that is thought to rely on the reuse of pre-existing neural systems for visual object processing (Dehaene and Cohen, 2007, 2011). From this perspective, the ability to combine spatially arranged visual elements, including morphemes in written language, into meaningful representations may reflect a domain-general mechanism of visual integration rather than a process specific to reading. For example, integrating related visual components, such as a wooden shaft and a sharp stone point to recognize a spear or a stone and a wooden stick to recognize a hammer-like tool, may reflect a general mechanism for constructing meaningful representations from distributed visual elements. Future studies could test this possibility by examining whether LAN-like effects are elicited during the integration of meaningful nonlinguistic visual components.

### 4.4 Implications for dual-route mechanisms of compound-word recognition across writing systems

The present findings provide important implications for refining the dual-route framework of Chinese compound-word recognition. First, the present study provides further electrophysiological evidence supporting the dual-route account of Chinese compound-word recognition and demonstrates that both whole-word orthographic and decomposition-related processing remain engaged despite substantial disruptions to the spatial configuration of constituent characters. Second, and more importantly, the present findings reveal that the flexibility of the two processing routes is not equivalent. Whole-word orthographic processing, as indexed by the ND250, was modulated by presentation orientation and character order, indicating that access to whole-word representations is closely associated with learned visual configurations of complete compounds. In contrast, decomposition-related processing, as indexed by the LAN, remained comparatively stable across different spatial configurations, suggesting that constituent morpheme representations are relatively robust to changes in character arrangement.

The present findings also raise important questions regarding whether similar spatially flexible dual-route mechanisms underlie compound-word recognition across different writing systems. Previous studies have shown that the ND250 can be elicited during visual word recognition in alphabetic languages (e.g., Proverbio et al., 2004; Strijkers et al., 2015), suggesting that lexical-level orthographic processing is not unique to Chinese but may represent a common feature of visual word recognition across writing systems. However, evidence regarding whether the ND250 reflects whole-word processing of compound words in alphabetic languages remains limited. As discussed by Huang et al. (2023), one possible reason for this limited evidence is that many alphabetic compounds (e.g., *grandmother*) contain a relatively large number of letters and may therefore be less likely to form compact visual representations. In contrast, Chinese compound words typically consist of only two characters, allowing them to form relatively compact orthographic representations. Future cross-linguistic research using shorter and more visually compact alphabetic compounds, such as *teacup* and *sunset*, may help determine whether ND250-indexed whole-word processing also contributes to compound-word recognition in other writing systems and whether ND250-indexed whole-word processing remains accessible when the arrangement of constituent morphemes is disrupted. Similarly, although LAN effects have been reported for morphologically complex words across various languages, including both alphabetic languages and Chinese (e.g., El Yagoubi et al., 2008; Krott and Lebib, 2013; Wei et al., 2023), whether the spatial stability of decomposition-related processing observed in the present study generalizes across writing systems remains unknown. Future studies examining morphologically complex words with disrupted morpheme arrangements across different languages may determine whether the robustness of LAN-related processing under spatial disruption reflects a general property of morphological decomposition or a characteristic associated with Chinese compound-word recognition.

### 4.5 Limitations and future directions

One limitation of the present study is that lexical and morphological characteristics of the Chinese compound words were not systematically manipulated. Chinese compounds can be classified into several structural types, such as modifier, coordinative, subject–predicate, verb–object, and verb/adjective–complement compounds, and previous research suggests that compound structure can influence Chinese compound-word recognition (e.g., Sun et al., 2024). Different compound structures may also differ in the relative extent to which the two processing routes contribute to their recognition, including the recognition of jumbled forms. Similarly, other lexical characteristics that influence Chinese compound-word recognition, including semantic transparency, word frequency, and age of acquisition, may also modulate the relative contribution of whole-word and decomposition-related processing. Future studies systematically manipulating these factors would help clarify how lexical and morphological properties influence the balance between the two processing routes during Chinese compound-word recognition, particularly for jumbled words.

A second limitation is that the present study focused exclusively on two-character compound words, which are the predominant word type in modern Chinese. For two-character words, jumbling necessarily involves a complete reversal of the two constituent characters (i.e., AB → BA), making this manipulation a specific case of character-order disruption rather than the more varied forms of internal transposition typically used in alphabetic languages (e.g., *family* → *filamy*). Although the present design provides a controlled way to examine character-order disruption in two-character words, future studies should investigate longer Chinese words, which would allow more varied forms of character transposition to be tested.

## 5 Conclusion

The present study showed that whole-word orthographic processing and processing associated with morphological decomposition can be engaged when Chinese compound words are presented in vertical and jumbled formats. However, the two processing routes showed different patterns of sensitivity to visuospatial organization: whole-word orthographic processing indexed by the ND250 was more sensitive to presentation format, whereas decomposition-related processing indexed by the LAN remained comparatively stable. These findings support dual-route accounts of Chinese compound-word recognition and suggest that whole-word and decomposition-related processing differ in their sensitivity to presentation orientation and character order.

## Data Availability

The raw data supporting the conclusions of this article will be made available by the authors, without undue reservation.
